# Young Children Are More Generous When Others Are Aware of Their Actions

**DOI:** 10.1371/journal.pone.0048292

**Published:** 2012-10-31

**Authors:** Kristin L. Leimgruber, Alex Shaw, Laurie R. Santos, Kristina R. Olson

**Affiliations:** Department of Psychology, Yale University, New Haven, Connecticut, United States of America; University College London, United Kingdom

## Abstract

Adults frequently employ reputation-enhancing strategies when engaging in prosocial acts, behaving more generously when their actions are likely to be witnessed by others and even more so when the extent of their generosity is made public. This study examined the developmental origins of sensitivity to cues associated with reputationally motivated prosociality by presenting five-year-olds with the option to provide one or four stickers to a familiar peer recipient at no cost to themselves. We systematically manipulated the recipient’s knowledge of the actor’s choices in two different ways: (1) occluding the recipient’s view of both the actor and the allocation options and (2) presenting allocations in opaque containers whose contents were visible only to the actor. Children were consistently generous *only* when the recipient was fully aware of the donation options; in all cases in which the recipient was not aware of the donation options, children were strikingly ungenerous. These results demonstrate that five-year-olds exhibit “strategic prosociality,” behaving differentially generous as a function of the amount of information available to the recipient about their actions. These findings suggest that long before they develop a rich understanding of the social significance of reputation or are conscious of complex strategic reasoning, children behave more generously when the details of their prosocial actions are available to others.

## Introduction

Human adults are unique in that they perform what appears to be an inordinate amount of generous behavior [1]–[4]; even more remarkably, empirical evidence indicates that hints of these prosocial tendencies are present even early in development. Research shows that infants as young as eight months of age willingly share toys with family members, peers, and complete strangers [5]–[7]. At 14 months of age, children will help an adult experimenter complete a goal [8] and will even take a cost to help others by the time they are 20 months of age [9]. Finally, between the ages of two and four, children begin to share resources with others voluntarily [10], even when those resources are easily monopolizable [11]–[12].

Why do children show prosocial behavior from such an early age? To date, prosocial behavior in children has primarily been explained in terms of intrinsic motivations such as empathy, other-regarding preferences, or a desire for fair outcomes (e.g., [8], [10], [13]–[21]). Under this view, children want to help others because they are motivated by that person’s need (see review: [22]). Other psychologists have suggested that prosocial behavior in infants and young children may also be driven by other motivations, such as wanting to prove oneself to be a useful and cooperative in-group member–i.e. wanting to present oneself favorably to others [23]. While a good deal of research has been done to investigate the role of intrinsic motivations on prosocial behavior in children, much less has been done to address the latter- what role, if any, do self-presentational motivations play in encouraging prosocial actions in young children? Unfortunately, because much of the research on prosocial behavior has been conducted using methods where a beneficiary and/or parent is present and aware of the child’s actions (e.g., [21], [24]), previous work cannot determine what role, if any, concerns with self-presentation may play in guiding this behavior.

To answer this question, it may be helpful to look at the factors associated with self-presentational motivations and prosocial behavior in adults in an effort to track the developmental trajectory of these tendencies. Recent research suggests that, at least for adults, prosocial actions stem in part from an implicit evolutionarily selfish motivation–to promote one’s reputation [25]–[31]. For the purposes of this paper, reputation is defined as information-based inferences about an agent’s character that may serve to inform others of the general nature of his/her possible actions in the future, thus leading to possible future reciprocation or punishment. This is reputation in its most basic instantiation, and research suggests that even young infants respond differently to agents who have good and bad reputations [32]–[33]. Although they may not be aware of it, adults appear to be selective about the situations in which they choose to act prosocially. Specifically, adults often maximize their performance of generous acts in situations in which there is an audience present to witness their actions [34]–[43].

Although the presence of an audience clearly affects people’s decisions about when to act prosocially, it is not clear that adults realize the extent to which audiences influence their behavior. Indeed, research suggests that people’s prosocial tendencies are impacted by audience cues that are even incidentally presented. Merely exposing people to eyespots or other subtle audience cues can increase prosociality in adults [44]–[47] (but see also [48]–[50]), even though people may not consciously realize their behavior is being influenced by these cues. The tendency to act more prosocially in the presence of subtle environmental cues that could possibly be perceived as an audience suggests that our reputational motivations may draw on the simplest and most evolutionarily old of cognitive mechanisms [51]. Indeed, these sorts of audience-dependent changes in behavior have been observed in other less cognitively-sophisticated species ranging from cleaner wrasses (*Labroides dimidiatus*) [52] to brown capuchin monkeys (*Cebus apella*) [53]. Overall, such cues– which we will refer to here as *audience cues–* appear to be extremely important in determining the extent of people’s prosocial behavior across a number of situations, although it is not clear that people are consciously considering their reputations in cases in which they are affected by these cues.

In addition to being sensitive to whether an audience is present, adults’ generosity also seems to be affected by a second set of cues: those related to the amount of information other parties have regarding the presence, absence, or degree of a prosocial action [54]. For example, consider a case in which a man is deciding how much to give to charity in the presence of an audience that will not know any details about the amount of money given. While the presence of audience cues would likely prompt him to make a donation, his reputation would not be further enhanced by performing an exceedingly generous action because the audience members would not know the full extent of his generosity. What is needed to maximize the impact of his generosity is the presence of a second set of reputationally relevant cues–which we will refer to as *transparency cues–* signals that indicate that people know which kind of action has occurred. As with audience cues, there is much work suggesting that adults are more generous when the specifics of their generosity are made available to others [35], [55]. For example, Andreoni and Petrie found a strong positive relationship between the amount of information made public about donor activity and the amount of money that was actually donated [35]. Conversely, in situations where recipients of generosity have limited information regarding the extent of an individual’s donation, people often behave in surprisingly *ungenerous* ways [56], [57]. Specifically, when the recipient of a donation is unaware of the details surrounding a possible act of generosity, people tend to act in ways that best serve their personal interest rather than that of the recipient. As in the case of audience cues, it is not clear that people are consciously thinking about reputation and intentionally changing their level of generosity in the face of transparency cues. Instead, people likely respond to transparency cues implicitly, using cognitive mechanisms developed over evolutionary time for the purpose of maximizing reputation.

Although there is reason to suspect that audience cues are conceptually distinct from transparency cues, both types of cues are often inextricably linked in real world situations. Indeed, the presence of transparency cues in non-experimental situations is often contingent on the fact that there is an audience likely to become aware of one’s actions. However, it should be noted that the two sets of cues are in no way mutually exclusive. As noted above, people are sensitive to audience cues (eyespots) in the absence of any true agents gathering information [46]. Additionally, even in the absence of any audience cues (when making a decision in a room by oneself) people are sensitive to whether other agents will be able to discover an ungenerous act [56].

Given that adults clearly modify their behavior in response to both audience and transparency cues, is it possible that young children do the same? Existing developmental literature suggests that children’s behavior more generally is influenced by the presence or opinions of others (see [58] for review). For example, around age three, children begin to engage in deceptive behavior (e.g., lying) to spare the feelings of others [59]–[63] and to cover their own indiscretions [64]–[68]. By the time children reach the age of five, they are able to understand the ways in which second-hand information like gossip can influence reputation [69]. Interestingly, it is not until later that children explicitly understand why people would want to present themselves to others in a specific way [70], are able to infer audience preferences on their own [71]–[73], and start to become skeptical about other people’s self-serving presentation biases [74]–[76]. Indeed, it is not until eight years of age that children begin to fully understand that other people may have self-presentational motives that affect the way they behave in the presence of others [77]. Overall, this pattern suggests that young children are sensitive to the opinions of others and modify their behavior accordingly long before they begin to grasp the concept of active reputation-management. As a result, it is possible that children’s own prosocial behavior may also be sensitive to audience and transparency cues years before they possess explicit knowledge of the social function of, and possibility for, strategic reputation management in others.

While evidence strongly suggests that children’s behavior is generally influenced by a desire to make a good impression in the eyes of others (e.g. [58]), no research to date has systematically addressed the role that audience and transparency cues play in mediating children’s prosocial tendencies. In fact, previous research looking at children’s prosocial tendencies has used a wide variety of methodologies with differing degrees of audience and transparency cues, making it difficult to compare across studies. For example, prosocial testing paradigms in children range from those in which children’s actions are anonymous even to experimenters [78], [79] to situations in which both the subject and the recipient of the prosocial act are present and fully aware of one another’s actions [80]–[82]. Furthermore, even when audience and transparency cues are experimentally manipulated, they are often confounded with other factors such as the in-group/out-group status of the recipient [12], [83]–[85], making the effects of these cues virtually indistinguishable from other factors. Overall, most authors fail to discuss or account for audience or transparency effects when interpreting levels of prosociality (but see: [86], [87]); as a result, there is still much to learn regarding whether young children’s prosocial decisions are sensitive to these cues.

The current study aimed to address this gap in the literature by directly testing the extent to which children’s prosocial behavior is affected by audience and transparency cues using a no cost allocation task. We did this by independently manipulating the visibility of the recipient (varying whether the recipient was occluded by a large opaque screen) and the transparency of the allocations to the recipient (presenting the allocations in either opaque containers or transparent containers). We chose to test five-year-olds because previous research has shown that children at this age can successfully represent the goals and beliefs of others (for review see [88]) and thus we knew that children of this age could understand what the recipient knew about different kinds of actions. Our question, then, was whether five year-olds’ decisions about whether to be generous would be sensitive to the amount of information available to the recipient. We tested pairs of children who were classmates and, thus, likely to interact with one another in the future. Given the notion that reputational motivations are strongly influenced by the likelihood for future reciprocation or punishment, pairing children with possible future collaborators was the truest way to test for such reputational concerns.

Overall, the logic behind the current study is as follows: if, in fact, prosocial behavior in children is largely intrinsically motivated, our subjects should act generously, regardless of if the recipient is aware of their actions. However, if children’s prosocial behavior is sensitive to extrinsic social factors, we should see variation in their allocation decisions relative to the different conditions. Specifically, if children are sensitive to audience cues, then five year-olds should act more generously on the allocation task when the recipient is visible than when the recipient is not. Additionally, if children are sensitive to the transparency of their actions when making prosocial decisions, then they should behave more generously when allocations are presented in transparent containers than when they are presented in opaque containers. Furthermore, if children’s prosocial decisions in this task are driven largely by extrinsic social factors, then our participants should act neutrally or even ungenerously when the recipient does not have knowledge about their actions. Overall, by manipulating both the visibility of the recipient as well as the information available to the recipient, we were able to gain insight into the ways in which audience and transparency cues influence children’s prosociality as well as the extent to which children’s prosociality is impacted by extrinsic factors.

## Methods

### Ethics Statement

The treatment of participants in studies described in this paper was in accordance with the ethical standards of the American Psychological Association. Participants’ parents provided written informed consent and all procedures were approved by the Human Research Protection Program at Yale University.

### Participants

We divided our participants into two separate roles: *actors* (who determined the allocations distributed) and *recipients* (who simply received the chosen allocations). Actors were 32 (16 female and 16 male, *M = *64.88 months; SD = 3.09) five year-old children from preschools in the New England area; each actor was paired with another child (18 female and 14 male, *M* = 64.59 months, SD = 7.83), who was designated the recipient. In total, 16 actors participated in the visible condition (9 female and 7 male, *M = *64.37 months, SD = 2.51) and 16 actors participated in the occluded condition (7 female and 9 male, *M* = 65.40 months, SD = 3.59). During the course of the experiment, the actor made all decisions regarding allocations, while the recipient merely watched and collected whatever resources the actor gave to her. Each child participated in only one session and the children never switched roles (i.e. the recipient never became the actor); both children were made aware of the fact that they would never switch roles, thus eliminating possible confounds associated with concerns about direct reciprocity within the experiment. Interaction between the participants was limited as they were instructed not to talk to one another. All children were paired with individuals from the same classroom and, therefore, were familiar with one another prior to testing.

### Materials and Procedure

**Figure 1:**
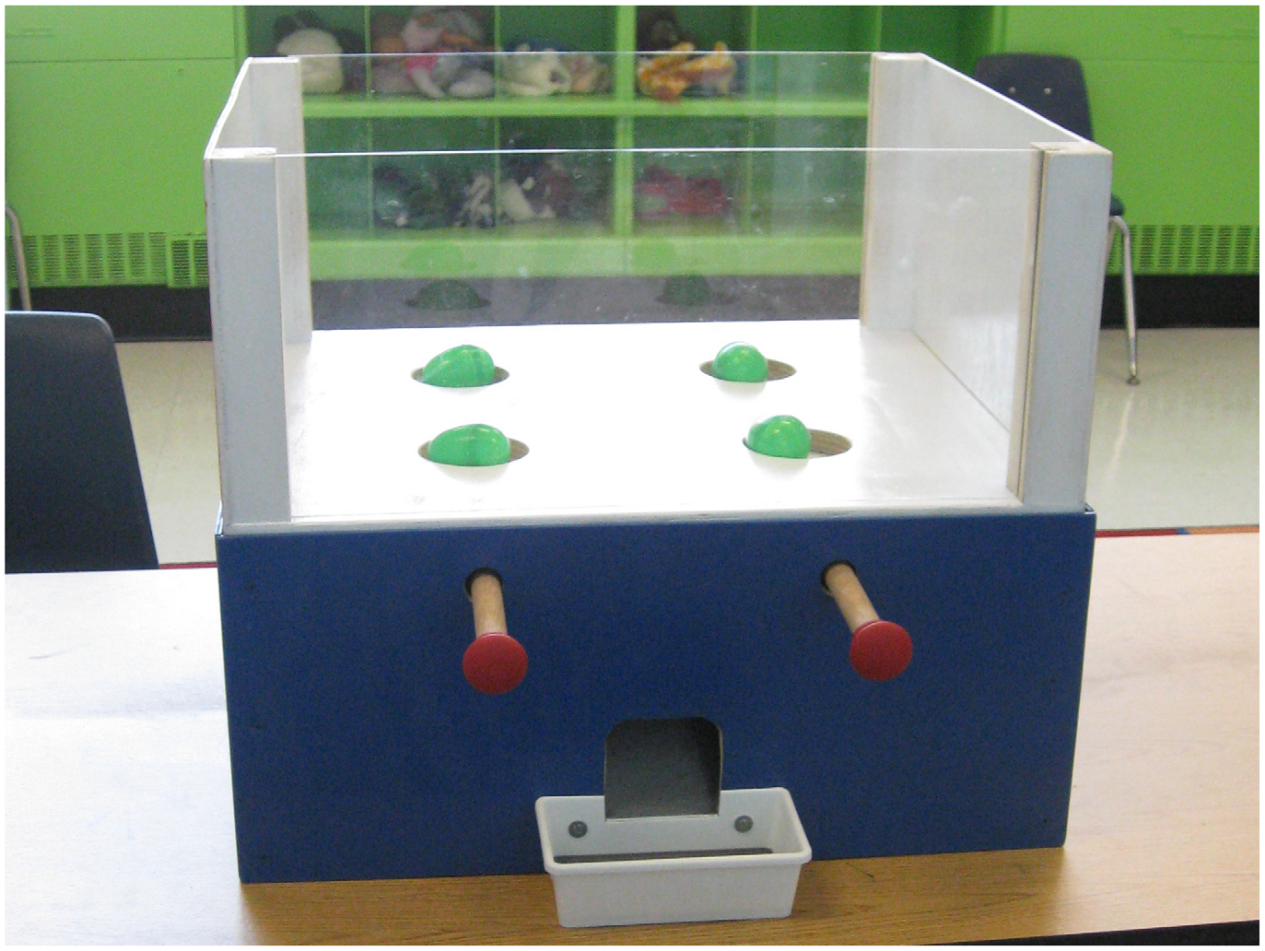
A view of the experimental set-up taken from the actor’s side of the apparatus. The actor was able to pull one of the two bars in order to deliver the allocations displayed on the top of the apparatus. Trial pictured is in the visible condition with opaque containers.

We constructed a testing apparatus that allowed the actor to choose between two allocation distributions; each distribution included an allocation for the actor and an allocation for the recipient (see Figure 1). In designing our apparatus, we took into consideration other devices that had previously been successful in testing children’s allocation decisions (e.g., [10], [89]). During test sessions, the apparatus was situated on a table between the actor and the recipient. The apparatus had two bars on its front that corresponded to the two distributions that sat in wells on the top of the apparatus. The actor always sat on the side with the bars while the recipient sat on the other side of the apparatus, directly across from the actor. By pulling the bar on her left, the actor could simultaneously distribute the left-side allocation distribution to herself and the recipient, with each child receiving the allocation directly in front of her. Conversely by pulling the bar on the right, the actor could deliver the right-side distribution. After allocations were delivered, both children placed their allocations, with assistance from the experimenters, in separate opaque cloth bags before the beginning of the next trial.

Both individuals were trained on the mechanics of the apparatus using differently colored “bouncy balls” and their understanding of the apparatus was confirmed through probe questions (e.g. “Pull the bar that gives the other girl a purple ball”; “What color ball will *you* get if you pull the left side?”). Overall, children seemed to find the apparatus quite intuitive and learned how to operate it almost immediately upon interacting with it. Testing commenced once both participants demonstrated an understanding of the outcomes associated with both sides of the apparatus; the training usually lasted about five minutes.

Allocations were distributed inside of plastic containers that were either opaque plastic or transparent plastic. When the containers were opaque, the allocations inside could not be seen, but when the containers were transparent the contents were visible. Children received different numbers of small stickers (either one sticker or four stickers) as allocations, and were told that each sticker was worth a point that they could cash in at the end of the test for a final grand prize. The grand prize for all participants was a large sticker of their choice; this served to eliminate possible social repercussions associated with one child getting an obviously superior prize than the other once they returned to class.

Each test session consisted of 16 trials. Two experimenters ran each session. The first experimenter presented the allocation options to the actor while the second experimenter sat next to the recipient and ensured that she paid attention throughout the session. During each trial, the first experimenter presented each container individually by showing the actor the contents and then allowing her to track the placement of the container in one of the four wells on top of the apparatus. The experimenter presented opaque containers in such a way that the actor, but not the recipient, could see the contents. Once the actor was aware of the container’s contents, the experimenter closed the container and placed it in the appropriate location. The order in which the containers were presented was randomized. Once all the containers were in place, the actor had a chance to pull one of the two bars in order to deliver either the left or right distribution. As soon as the actor committed to a distribution and pulled a bar, the experimenter removed the remaining two containers to eliminate additional pulling. Each test session consisted of eight trials using the opaque containers and eight trials using the transparent containers; blocks of opaque/transparent containers were broken up into alternating groups of four trials. Subjects were assigned to one of six different counterbalanced distribution orders.

To ensure that the actor’s choices were motivated by the recipient’s allocation and not their own, the actor’s allocation was constant within trials; actors always received the same number of stickers regardless of which distribution they chose. Research shows that young children often tend to act in ways that maximize their own self-interest when presented with sharing tasks in which they stand to gain from *not* acting prosocially [12], [83], [90]. For this reason we opted for a straightforward allocation task in which the actor’s own allocation options were equal within each trial.

While the allocation options for the actor were kept constant within trials, the allocation options for the recipient were varied. Each trial consisted of one of four different conditions, with each kind of trial presented four times during the test session (twice in opaque containers and twice in transparent containers). For simplicity, distributions will be denoted as a pair, with the actor’s allocation denoted first and the recipient’s allocation second. For example, if the actor chose a distribution in which she got one sticker and the recipient got four stickers, this will be represented as (1,4).

Precautions were taken to eliminate social cueing from the recipient, assure that the actor’s choices could not be seen in the relevant conditions, and minimize the possibility for conflict or punishment once the children returned to the classroom. First, participants were told the experiment was a “quiet game” and asked not to communicate with one another during the duration of the test session; this eliminated any verbal and non-verbal cuing that took place and freed the actor from having to answer questions about her allocation choices. Second, both children were asked to place their allocations into separate, opaque cloth bags after each trial to minimize distractions and also to reduce the ability of the recipient to directly compare her own allocation to the one received by the actor. At the end of the test session, children were taken to opposite ends of the testing room with different experimenters to count their stickers. All participants were told they had “just enough sticker points to get a big sticker,” which removed any evidence of inequity that may have occurred during the experiment. Taken together, these precautions reduced extraneous social factors that may have caused the actor to behave in a more prosocial manner for reasons other than those for which we were testing.

Finally, to examine the influence of audience cues on allocation decisions, we varied the visibility of the recipient between subjects. Half of our actors were tested in a *visible* condition, in which the actor and recipient were able to maintain visual contact throughout the test session. The other half of actors were tested in an *occluded* condition, in which a large, opaque piece of foam-board was placed into the apparatus on the end closest to the recipient, eliminating the recipient’s visual access to the actor and the allocation options for the duration of the test session.

Each test session consisted of eight critical trials and eight non-critical trials that were presented in a counterbalanced order; as a result, there is no reason to believe that participation in non-critical trials influenced children’s behavior in critical trials. Critical trials were the focus of data analysis, as they were the only trials in which the actor had a choice between a more and less generous allocation for the recipient. Non-critical trials were included in test sessions to ensure that the recipient received a mix of one and four sticker allocations, and therefore, was less able to deduce what the actor’s options had been on any given trial.

Critical trials were comprised of two types of choices: *High Value Choice* trials and *Low Value Choice* trials. In *High Value Choice* trials, the actor received a high-value allocation (four stickers) for herself and chose between an allocation of four stickers (4,4) or one sticker (4,1) for the recipient; the (4,4) distribution was the prosocial (more generous) choice. The actor received two High Value Choice trials in opaque containers and two high value choice trials in transparent containers over the course of the test session. In *Low Value Choice trials,* the actor received a low-value allocation (1 sticker) for herself and chose between an allocation of four stickers (1,4) or one sticker (1,1) for the recipient; the (1,4) distribution was the prosocial (more generous) choice. The actor received two Low Value Choice trials in opaque containers and two low value choice trials in transparent containers over the course of the test session.

Non-critical trials were also comprised of two different types of choices: *All Equal* trials and *Unequal Identical* trials. In *All Equal* trials, both the actor and recipient received the same allocation and that allocation was equal; the actor had the choice between distributions of either (1,1) vs. (1,1) or (4,4) vs. (4,4). The actor received one (1,1) trial and one (4,4) trial in opaque containers and one (1,1) trial and one (4,4) trial in transparent containers over the course of the test session. In *Unequal Identical* trials, the actor received either one sticker or four stickers and the recipient received the oppositely valued allocation. Specifically, the actor always received one sticker while the recipient always received four stickers, (1,4) vs. (1,4), or vice versa (4,1) vs. (4,1). The actor received one (1,4) trial and one (4,1) trial in opaque containers and one (1,4) trial and one (4,1) trial in transparent containers over the course of the test session.

## Results

Preliminary tests showed no significant effects for or interactions with gender so the authors collapsed across these groups for the remainder of the analyses; all *p*s>.10. Within each test session, the actor had eight critical trials: four were high-value choices (two trials in transparent containers, two trials in opaque containers) and four were low-value choices (two trials in transparent containers, two trials in opaque containers). We were interested in how often the actor chose the prosocial option (four stickers) over the antisocial option (one sticker).

**Figure 2:**
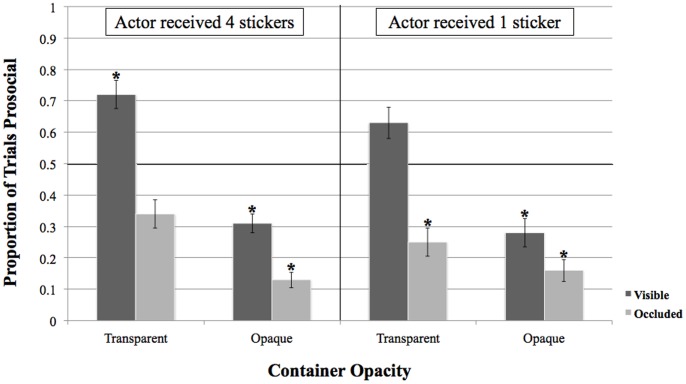
Main Findings. Mean proportion of prosocial giving (distributing four stickers rather than one sticker to the recipient) by visibility (visible versus occluded), container opacity (opaque versus transparent), and actor’s allocation value (one sticker versus four stickers).

A mixed-model repeated-measures ANOVA was conducted with visibility (recipient visible, recipient occluded) as a between-subjects factor and actor’s personal allocation (one sticker, four stickers) and container opacity (transparent, opaque) as within-subjects factors (Figure 2). We found a main effect of recipient visibility, indicating that actors were more prosocial when the recipient was visible (*M = *48%) than when the recipient was occluded from view (*M* = 22%), *F*(1, 30) = 15.32, *p*<.001, suggesting that the lack of a visible audience led to more ungenerous behavior in this task. In addition, we observed a main effect of opacity; actors were significantly more ungenerous when allocations were presented in opaque containers (*M* = 22%), than when they were presented in transparent containers (*M* = 48%), *F*(1,30) = 18.77, *p*<.001, suggesting that the amount of information available to the recipient regarding the allocation options also played a role in children’s allocation decisions. Lastly, the main effect of the actor’s own allocation value shows no overall significant difference in prosocial behavior regardless of whether the actor received four stickers (*M* = 38%) or one sticker (*M* = 33%), *F*(1,30) = 0.72, *p* = .402.

Furthermore, the interaction between actor-recipient visibility and container opacity was nearing significance, *F*(1,30) = 3.18, *p* = .085. Paired t-tests indicate that children in visible condition were more generous when the containers were transparent (M = 67%) than when the containers were opaque (M = 30%), *t*(1,15) = 3.77, *p* = .002. Similarly, children in the occluded condition were more generous when the containers were transparent (M = 30%) than when they were opaque (M = 14%), *t*(1,15) = 2.18, *p* = .046.

We next examined whether allocations in each of these four primary conditions differed from chance using one-sample t-tests. Actors distributed allocations more generously than would be expected by chance (50%) on trials in which the recipient was visible and the allocations were presented in clear containers (*M = *67%), *t*(1,15) = 2.20, *p* = .044. In contrast, actors selected *ungenerous* options more often than expected by chance (50%) when the recipient was visible and allocations were in opaque containers (*M = *30%), *t*(1,15) = 3.11, *p* = .007, when the recipient was occluded from view but allocations were in transparent containers (*M* = 30%), *t*(1,15) = 3.11, *p* = .007, and when the recipient was occluded from view and allocations were opaque containers (*M* = 14%), *t*(1,15) = 7.90, *p*<.001.

No interactions involving the actor’s personal allocation (1 vs. 4 stickers) approached significance, all *p*s>.30. However, for exploratory purposes, supplemental one-sample t-tests were performed to examine whether children were systematically more or less generous than chance in each of the eight critical trial types. In the most open condition- the case where the containers were transparent and the recipient was visible- actors were more likely than chance to provide four stickers (the generous option) to the recipient when they received four stickers themselves (*M = *72%), *t*(15) = 2.41, *p* = .029, but not when they received only one sticker (*M* = 63%), *t*(15) = 1.29, *p* = .216. In contrast, when the recipient was visible but allocations were presented in opaque containers (and, thus, hidden from the recipient’s view) actors were significantly more likely to give only one sticker (the ungenerous option); this was true regardless of whether the actor received four stickers (*M* = 31%), *t*(15) = 3.00, *p* = .009, or one sticker (*M* = 28%), *t*(15) = 2.41, *p* = .029, themselves.

When visibility between the actor and the recipient was occluded and, thus, the recipient could not see either the actor or the allocation distributions, overall rates of generosity were very low. When allocations were presented in transparent containers, actors were significantly more likely than chance to distribute one sticker to the recipient when they received one sticker (*M* = 25%), *t*(15) = 2.74, *p* = .015, but not when they received four stickers, (*M* = 34%), *t*(15) = 1.78, *p* = .096, themselves. Additionally, when visibility between the actor and the recipient was occluded and allocations were presented in opaque containers, rates of giving four stickers were the lowest (and therefore rates of giving one sticker were the highest) and therefore most different from chance; this was true when actors received four stickers (*M* = 13%), *t*(15) = 6.71, *p*<.001, or one sticker (*M* = 16%), *t*(15) = 4.57, *p*<.001, themselves. Finally, the fact that children behaved in patterns that differed significantly from chance for all but one of these conditions suggests that confusion about the experimental paradigm and the apparatus can be ruled out as possible explanations for our pattern of results.

## Discussion

The present results provide evidence that five-year-old children’s generosity is heavily influenced both by the presence of a visible audience and by the transparency of their actions. Although our participants donated allocations generously when the recipient was visible and the allocation options were transparent, children became systematically less generous if one or both of these factors were absent. Overall, actors were consistently more generous when the recipient was visible than when she was occluded from view, suggesting the important role audience cues play in children’s prosocial behavior. However, even when actors could see the recipient, children were systematically *less* generous when the allocations were presented in opaque containers than when the allocations were presented in transparent containers, that is, when the recipient was unaware of the actor’s allocation options. This suggests that children’s prosocial decisions are indeed motived by extrinsic factors and that the transparency of their actions strongly influences decisions regarding when to act prosocially, even in the presence of an audience.

One striking aspect of our results is that children were considerably ungenerous in our task. Indeed, children only showed consistently prosocial behavior in our study in the condition when they could see the recipient and their allocations were fully visible; in all other conditions, children were statistically ungenerous, giving the recipient the smaller amount of stickers. It is worth noting that this level of prosociality is lower than what one might have initially expected based on previous developmental work testing children’s sharing and allocation of resources, (e.g., [78], [79], [83]–[85], [87], [91]). Such previous work has generally reported that children behave rather generously, however it is hard to decipher the role that audience and transparency cues may have played in previous studies. As such, previous studies that observe high levels of generosity may have inadvertently included the same audience and transparency cues that we observe contribute to high rates of prosociality. Therefore, it is difficult to make direct comparisons between levels of generosity observed in our study and those reported in previous research. However, given the strong influence audience and transparency effects had on prosocial behavior in our study, it is our hope that researchers will take these factors into account much more often when designing studies examining prosocial behavior in the future.

One additional explanation for the low rates of giving we observed in our study is that children may have been unintentionally primed to think of our experiment as a competition. When describing our study to children, we referred to our allocation task as a “game” and told children that they could use their accrued stickers as tokens to get a prize. Thinking about our task as a game in which tokens were going toward a prize may have put children in a competitive mindset, thus making them want to try to accrue more tokens than the recipient. Indeed, even adults will work harder for a prize in cases where they stand to get relatively more tokens, even if (as in our task) the tokens cannot be kept and are used to get a pre-determined prize [92]. We also know from previous work that being placed in a competitive mindset causes children to place a larger value on getting more than others (see for example, [93]). It is possible, then, that children behaved more ungenerously on our task because they thought the game was a competitive one.

The potential for children to have construed our task in a competitive framework highlights another potential framing of our results. We initially interpreted our findings as showing evidence that children exhibit more generosity when transparency and audience cues are present. However, our results are also consistent with the possibility that transparency and audience cues work the opposite way– rather than increasing children’s generosity, such cues might instead inhibit children’s *ungenerous* behavior.

Under either framing of the results, the upshot is the same: children modify their behavior in response to the presence of audience and transparency cues. When audience and transparency cues were present, children were substantially more willing to give resources to another recipient. Competitive motives may have reduced children’s overall rates of generosity in our study, but children restricted these competitive impulses in cases where the recipient could see them and when the recipient would know if she was given less. We further found that both the presence of an audience and transparency cues independently influenced children’s behavior. Based only on the present results, it is difficult to know whether children acted more generously in the presence of audience and transparency cues, or whether they inhibited their ungenerous tendencies in the presence of such cues.

Our results also leave open the question of *why* children exhibit more generous behavior in the presence of audience and transparency cues. One possibility is that children behave more generously because seeing the recipient allows them to have more empathy for that individual [44], [45]. Another possibility is that children dislike experiencing a negative response when the recipient receives bad payoffs [94]; under this view, children would be more generous when they can see the recipient because they are trying to avoid feeling bad because they have had to witness another individual’s negative reactions. However, the fact that children in our study acted ungenerously even when they could see the recipient’s reaction when allocations were presented in opaque containers suggests that neither of these explanations can account entirely for our results. Given that we cannot unequivocally rule out either of these possible explanations, further research is needed to fully elucidate the mechanisms by which audience and transparency cues affect children’s generosity.

Overall, our results illustrate that children’s prosocial decisions are sensitive to both audience and transparency cues years before children seem to have an explicit understanding of the concept of reputation (for a review see [58]), and that prosocial motivations in young children are not influenced solely by intrinsic motivations. While existing evidence shows that children do not begin to understand self-promotional reputation enhancement until around eight years of age [71], [72], [77], our findings reveal that children as young as five years of age behave in ways consistent with adult patterns of prosociality in response to audience and transparency cues. As a result, our findings suggest that the sensitivity to audience and transparency cues that are thought to be key to reputation enhancing prosociality in adults may have deeper developmental roots than researchers have previously suspected.
